# A Case Series of Surgically Treated Distal Radius Fractures: Implant Costs and Their Effect on Patient Outcomes

**DOI:** 10.5435/JAAOSGlobal-D-23-00026

**Published:** 2023-07-07

**Authors:** Stephen A. Doxey, Fernando A. Huyke-Hernández, Jennifer L. Robb, Deborah C. Bohn, Brian P. Cunningham

**Affiliations:** From the Department of Orthopaedic Surgery (Dr. Doxey, Mr. Huyke-Hernández, Dr. Cunningham), Park Nicollet Methodist Hospital, St. Louis Park, MN (Dr. Doxey, Huyke-Hernández, and Dr. Cunningham); the Department of Orthopaedic Surgery(Dr. Doxey, Huyke-Hernández, Dr. Robb, Dr. Bohn, Dr. Cunningham), TRIA Orthopaedic Institute, Bloomington, MN (Dr. Doxey, Huyke-Hernández, Dr. Robb, Dr. Bohn, and Dr. Cunningham); and the Department of Orthopaedic Surgery, University of Minnesota, Minneapolis, MN (Dr. Bohn).

## Abstract

Distal radius fractures (DRFs) are common injuries, warranting a need to analyze the cost of treatment. The purpose of this study was to analyze the effect of implant costs on patient-reported outcomes in DRFs.

**Methods:**

A PRO registry was retrospectively reviewed for isolated, surgically treated DRF patients. A total of 140 patients met criteria to be included in this study. Implant cost was obtained from the chargemaster database.

**Results:**

The average total implant cost was $1,289.67 ± 215.32. The average Patient-rated Wrist Evaluation scores preoperatively, at 6 weeks, and at 12 weeks were 70.8 ± 20.1, 36.6 ± 21.1, and 22.8 ± 18.0, respectively. No statistically significant relationships were observed between cost and Patient-rated Wrist Evaluation scores at 6 weeks or 12 weeks (r = −0.05, *P* = 0.59; r = −0.04, *P* = 0.64, respectively). Implant costs were shown to not be affected by fracture complexity (AO/OTA classification: 23A = $1,335.50, 23B = $1,246.86, and 23C = $1,293.14).

**Discussion:**

The total cost of implants did not influence patient outcomes indicating that patients receive no additional benefit from more costly constructs.

Approximately 634,000 distal radius fractures (DRFs) occur in the United States (US) each year, making them one of the most common fractures.^1^ They are second only to hip fractures in the geriatric population.^1^ Medicare spends $385 to $535 million dollars annually on DRFs in the elderly population alone.^2,3^ With this substantial economic burden and rising cost of health care in the United States, there has been increasing focus on providing high-value patient care. Value is defined as the ratio of outcomes to cost.^4,5,6,7,8,9^ Outcomes in DRF patients can be defined using patient-reported outcome measures (PROMs) such as the Patient-rated Wrist Evaluation (PRWE). PROMs ensure that decreases in cost do not negatively affect patient care.^10^

DRF treatment historically consisted of casting or percutaneous fixation, which are relatively low-cost. However, costs have markedly increased with the introduction of the volar locking plate (VLP). Surgeon preference for open reduction and internal fixation (ORIF) of DRFs has also increased in recent years.^1^ In 2007, Medicare spent $170 million on DRF treatment, with ORIF accounting for 32% of expenses.^11^ Previous literature has analyzed the difference in cost between ORIF and closed reduction and percutaneous pinning (CRPP) as well as between surgical and nonsurgical treatment, but to date, there has been no evaluation of the cost of VLPs and their relationship to patient-reported outcomes (PROs).^12,13^ As implants have been found to account for an average of 43 to 50% of total procedure costs and even up to 87% in some orthopaedic procedures, there is a need to determine cost-effective use of implants in DRF treatment.^14^ The value of other surgical procedures has been analyzed in the setting of ankle fractures^15^ and rotator cuff tears,^16^ finding that increased implant costs do not lead to better patient outcomes. No study to date has analyzed the relationship between implant costs and PROs in DRF patients.

The goal of value-based care is to provide improved outcomes at lower costs. The primary purpose of this study was to evaluate the relationship between total implant cost and PROs in DRFs. The secondary purpose was to evaluate the effect of vendor and fracture severity on total construct cost. It was hypothesized that increasing implant costs do not lead to increased PRWE scores. The primary outcomes of this study were PRWE scores and cost of implants.

## Methods

After approval by the Institutional Review Board, a retrospective review of a prospectively collected PROM database was done for patients from 2014 to 2021. A total of 1,048 patients with a DRF were identified within a single healthcare system. Inclusion criteria consisted of patients having a DRF treated with a VLP and complete PRWE data. Patients were excluded if they were skeletally immature, sustained polytrauma, sustained an open fracture, or had missing PRWE scores. DRFs treated nonsurgically or with surgical techniques other than a VLP were also excluded.

Patient records were accessed for baseline data consisting of age, sex, AO/OTA fracture classification, injury of the dominant hand, a preexisting diagnosis of depression or anxiety, smoking history, and treatment facility. PRWE scores at preoperative baseline and at 6-week and 12-week follow-up were extracted from the institutional PROM database. The PRWE instrument consists of two portions: pain and function. Both portions are scored out of 50 and added together for a total of 100, with 100 indicating severe pain and disability and 0 indicating no pain nor disability (Table 1).^17^ Implant data were extracted from patient records and included plate model and medical device company, screw type, peg type, and number of screws and pegs used. To obtain implant cost, model numbers were cross-referenced across the system's charge master database.

**Table 1:** Aspects of the Patient-rated Wrist Evaluation

Aspects of the Patient-rated Wrist Evaluation
Pain^a^
At rest
Doing a task with repeated wrist/hand movement
Lifting a heavy object
When it is at its worst
How often do you have pain?^b^
Function^c^
Specific activities
Fasten buttons on your shirt?
Cut meat (or vegetables) using a knife?
Turn a door knob with your affected hand
Use your affected hand to push up from a chair?
Carry a heavy object in your affected hand?
Use bathroom tissue with your affected hand?
Usual activities
Personal care activities (such as dressing/washing)
Household work (such as cleaning or maintenance)
Work (your job or other work)
Recreational activities

a The average amount of pain reported on a 10-point Likert scale with 0 indicating “no pain” and 10 indicating “worst ever.”

b Reported on a 10-point Likert scale with 0 indicating “never” and 10 indicating “always.”

c The amount of difficulty experienced performing these tasks on a 10-point Likert scale with 0 indicating “no difficulty” and 10 indicating “unable to do.”

Data were analyzed using SPSS software, version 28 (Chicago, IL). Descriptive statistics are provided for continuous and categorical variables. Frequencies for total implant costs were graphed. A Pearson product-moment correlation was done to assess the relationship between total implant cost and PRWE score and score changes at all time points. To evaluate the relationship between total implant cost and VLP medical device company, a Spearman rank order correlation was done.

## Results

**Figure 1:**
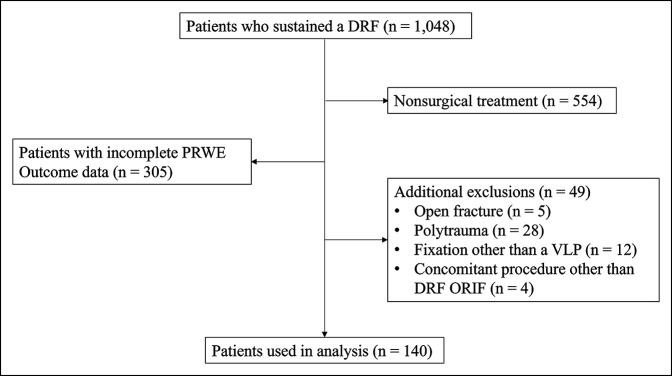
Flow diagram of inclusion and exclusion criteria for final analysis. DRF = distal radius fracture, PRO = patient-reported outcome, PRWE = Patient-rated Wrist Evaluation, VLP = volar locking plate, ORIF = open reduction and internal fixation.

A total of 140 patients met criteria for analysis (Figure 1). The average age was 59 years, and 85.7% of patients were female. Approximately 53.6% of patients injured their dominant hand. Only 35.7% of patients presented to a level 1 trauma center. Most of the patients were nonsmokers (67.9%). Those diagnosed with depression were commonly diagnosed with anxiety as well (17.1%). Most of the patients presented with an AO/OTA 23C fracture (66.4%) (Table 2).

**Table 2:** Demographics of Patients With Distal Radius Fractures Undergoing Surgical Fixation

Demographic Factor	(N = 140)^a^
Age	59.0 ± 13.7
Sex	
Male	20 (14.3%)
Female	120 (85.7%)
Dominant hand injured	75 (53.6%)
Treated at a level 1 trauma center	50 (35.7%)
Mental health diagnosis	
Depression	22 (15.7%)
Anxiety	8 (5.7%)
Both	24 (17.1%)
Smoking history	
Nonsmoker	95 (67.9%)
Current	34 (24.2%)
Former	11 (7.9%)
Fracture classification	
23A	34 (24.3%)
23B	13 (9.3%)
23C	93 (66.4%)
Preoperative PRWE	70.8 ± 20.1
6-week PRWE^a^	36.6 ± 21.1
12-week PRWE	22.8 ± 18.0

a Continuous data reported as mean ± standard deviation. Categorical data reported as N/n (%).

PRWE = Patient-rated Wrist Evaluation

**Figure 2:**
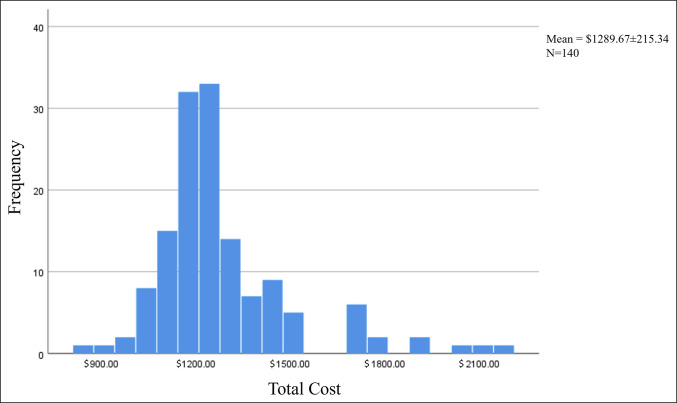
Frequency and cost of implants for distal radius fractures. This figure demonstrates the total cost of a fixation construct on the x-axis with the frequency of fixation cost shown on the y-axis. The mean price was found to be $1289.67 ± 215.24, and the sample size was 140.

**Figure 3:**
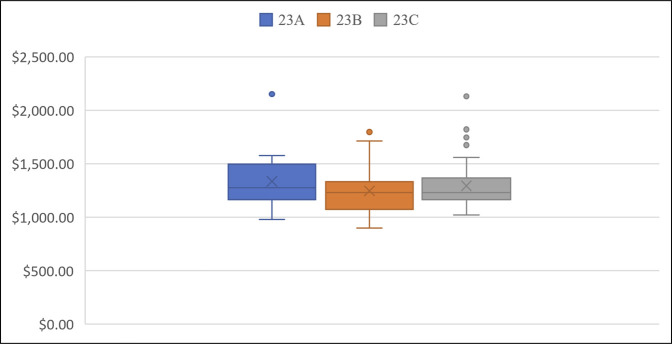
Total construct cost by fracture classification. This is a box and whisker plot showing differences in cost by AO/OTA fracture classification. 23A = extra-articular, 23B = partially intra-articular, and 23C = complete intra-articular.

**Figure 4:**
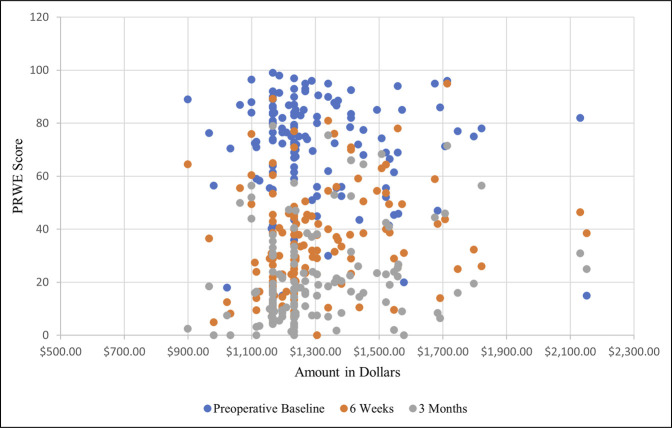
Graph showing the patient outcomes compared with total cost. The x-axis represents total cost, and the y-axis represents PRWE (Patient-rated Wrist Evaluation) scores. The different colors represent varying periods of time, with blue indicating preoperative baseline, orange meaning 6-week follow-up, and gray representing 3-month follow-up.

The VLP design consisting of three holes in the proximal segment and seven in the distal segment was the most commonly used (n = 98, 70%). Nonlocking screws used on average were 3.1 (range: 0-8) per plate. An average of 7.1 (range: 0-9) locking screws (variable angle and nonvariable angle) and 6.1 (range: 0-9) locking pegs (variable angle and nonvariable angle) were used per plate. The average total implant cost ranged from $898.72 to $2151.36, averaging $1,290 (Figure 2). The total cost was not found to be related to the medical device company used (r_s_ = −0.09, *P* = 0.29). The number of patients and range of total construct costs per vendor are as follows: DePuy Synthes (n = 17, $1289.12-$1797.18), Arthrex (n = 32, $1062.83-$1821.00), Stryker (n = 1, $2130.87), and Zimmer Biomet (n = 90, $898.72-$2151.36). The cost by AO/OTA fracture classification 23A, 23B, and 23C was found to be $1335.50, $1246.86, and $1293.14, respectively (Figure 3). The average PRWE scores preoperatively, at 6 weeks, and at 12 weeks were 70.8 ± 20.1, 36.6 ± 21.1, and 22.8 ± 18.0, respectively. No statistically significant correlations were observed between construct cost and PRWE scores at 6 weeks or 12 weeks (r = −0.05, *P* = 0.59; r = −0.04, *P* = 0.64; respectively) (Figure 4). No significant correlations were observed between total construct cost and the change in PRWE scores from baseline to 6 weeks, baseline to 12 weeks, or 6 weeks to 12 weeks (r = 0.05, *P* = 0.55; r = 0.045, *P* = 0.60; r = −0.02, *P* = 0.83, respectively).

## Discussion

Patient outcomes can be influenced by a variety of factors inherent to the patient, injury, or treatment. The purpose of this study was to determine whether more costly VLP construct costs lead to better patient outcomes. It was hypothesized that increasing implant costs do not positively influence PROs. This study found that follow-up PRWE scores and the changes in PRWE scores do not correlate with the total cost of fixation. These results suggest that more expensive VLP fixation constructs do not provide better patient outcomes. The total construct costs were similar among AO/OTA fracture types, suggesting that fracture severity does not influence the number and type of screws used in VLPs. The implant vendor used did not correlate with total cost, indicating that VLPs are similar in price among the companies in this study.

Despite the increasing prevalence of surgically treated DRFs, the optimal treatment modality is still unknown. There is question as to which DRFs need surgical fixation. The literature has shown low complication rates and good outcomes with the use of VLPs.^1,18^ Chung et al.^19^ demonstrated successful outcomes using nonsurgical treatment even in the presence of a suboptimal reduction. Arora et al.^20^ conducted a randomized controlled trial between ORIF with a VLP and nonsurgical treatment. At 1 year, they found no difference in range of motion (ROM), pain, or the PRWE and the Disabilities of Arm, Shoulder, and Hand questionnaire (DASH) scores. A meta-analysis of five randomized control trials with 216 to 613 patients (depending on the variable analyzed) showed similar results comparing ORIF with nonsurgical management in older adults and found that surgically treated patients had better DASH scores and grip strength, yet no difference in pain, complication rates, or ROM at 1- to 2-year follow-up.^21^ Controversy regarding the DRF method of treatment continues to exist and ultimately has varying financial implications depending on the chosen modality due to additional costs of the procedure, implants, and recovery.

Previous literature has analyzed the cost of ORIF compared with other types of treatments. Nandyala et al. randomized 40 patients to undergo CRPP or ORIF for treatment of their closed, displaced, unstable DRF. They found that ORIF costs were 2.7 times greater than CRPP in the initial perioperative period because of operating room time and implant costs. By 1 year, the difference in cost was reduced to 1.6 times greater than CRPP. They concluded that the cost balance is due to CRPP-treated patients needing additional surgeries and additional physician and therapy appointments.^12^ Toon et al. studied 60 patients with closed intraarticular DRFs treated with ORIF or nonsurgical management (32 vs. 28, respectively).^13^ They showed that although overall functional outcomes were not markedly different at 1-year follow-up (average DASH score was 16.2 in ORIF and 16.1 in nonsurgical), ORIF increased costs by 37-fold compared with nonsurgical treatment.^13^ These studies show that although outcomes did not differ markedly between treatment modality, there was a difference in costs. Similar to these previous findings, this study demonstrated that treatment cost varies between patients without notable differences in outcomes. Specifically, this study analyzed surgical patients and the specific type and number of plates and screws. Through the use of PROs, surgeons can inform themselves on how to minimize cost and maximize the value of treatment for their patients.

PROMs are increasingly being used to evaluate the effectiveness of treatment. PROMs along with costs are key components of the value equation. The concept of value has been assessed in the treatment of other injury patterns outside of DRFs. In rotator cuff repair, it was shown that there was no association between the total cost of care and the patient outcomes.^16^ Although it only evaluated implant costs, this study similarly shows that rising costs do not lead to better outcomes. Wise et al^16^ also demonstrated that the highest-value procedures in rotator cuff repair were in patients with a single-tendon arthroscopic repair, whereas open repair procedures demonstrated the lowest value. McCreary et al. analyzed the value in ORIF of ankle fractures and found that inpatient stay was associated with decreased value of treatment.^15^ In this study, the authors elected to use the PRWE because it has been shown to be a validated and reliable instrument for evaluating outcomes in patients with DRFs.^22^ This study demonstrated an average downward trend in PRWE scores from injury to 12-week follow-up, indicating an overall improvement since treatment. However, no correlation was found between the implant cost and PRWE improvement, suggesting that less costly implants could be used without hindering patient outcomes and thereby maximize procedural value. Additional investigation should include more factors than solely implant costs to evaluate the complete episode of care for DRFs, as has been done in previous value literature.^4,15,16,23^

**Figure 5:**
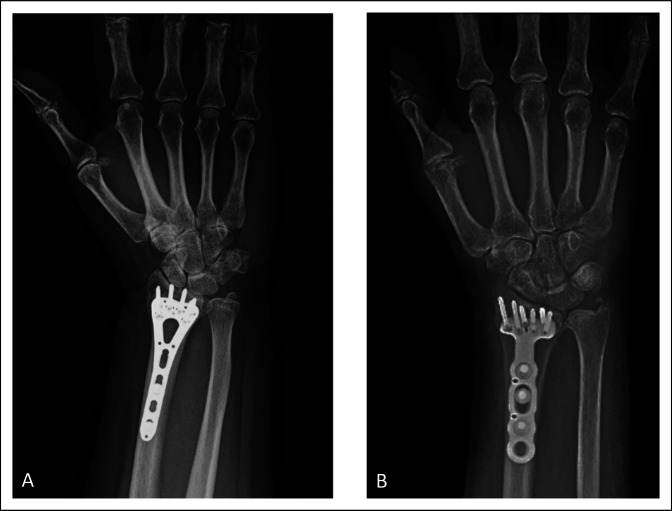
Radiographs showing the distal radius fracture fixation with differing implant vendor plates. **A,** This is a fracture treated with a DePuy Synthes plate that costs $1746.15. Their PRWE score at baseline was 77 and at 12 weeks was 16. **B,** This is a fracture treated with a Zimmer Biomet plate that costs $1165.72. Preoperative PRWE was 80 and at 12 weeks was 38. Both patients sustained an AO/OTA 23C type fracture.

**Figure 6:**
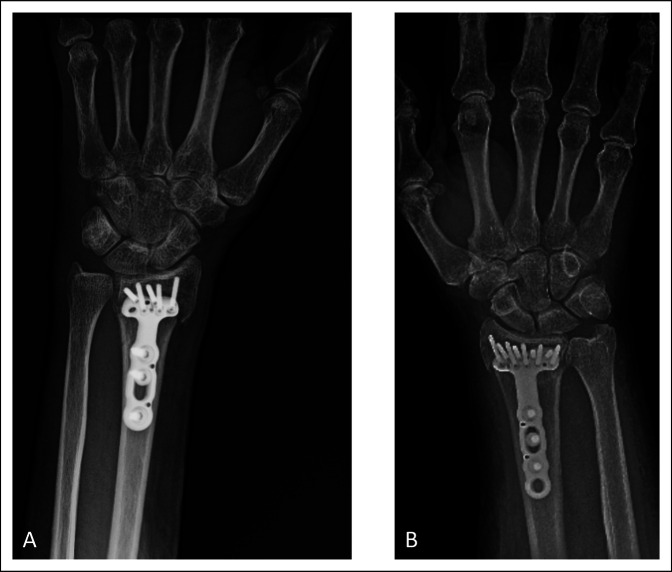
Radiographs showing the distal radius fracture fixation with differing constructs within the same vendor. Both of these fractures were treated with Zimmer Biomet plates. **A,** This construct had five distal locking pegs that cost $1058.08. Their PRWE baseline score was 96.5 and at 12 weeks was 56.5. **B,** This construct had nine distal locking pegs that cost $1325.08. Their PRWE score at baseline was 55.5 and decreased to 21.5 at 12 weeks. Both patients presented with AO/OTA 23C type fractures.

As reimbursement models shift from fee-for-service to value-based care, there is a growing need for continued research of value in orthopaedics. Currently, there is minimal research regarding orthopaedic procedures using the value equation, although it is pertinent to provide more effective and efficient care to patients. Surgeons do not have the ability to set the price of implants used, but they can opt to use less costly plates and screws to optimize the value equation. In ankle fractures, it has been shown that the use of cannulated screws in fixation leads to higher total fixation costs.^10^ In DRFs, variable angle and locking screws typically are more expensive when compared with cortical screws. As the fracture permits, surgeons can potentially decrease the overall cost by choosing to use different implants or decreasing the number of locking screws used in the fixation construct. The surgeons in this study tended to fill most if not all screw holes in the VLP, regardless of fracture pattern. This may have led to the similar results stratified by OTA classification. Adjusting the number of screws to fracture pattern is a potential way to decrease cost while maintaining outcomes as has been shown in other orthopaedic procedures.^24^ Future studies are needed to assess the relationship between number of screws used in fixation and patient outcomes in DRFs. Figures 5 and 6 show examples of differences in cost between implant vendors and number of screws used within the same vendor. Currently, low-cost, generic implants are being developed, which can be another potential opportunity to effectively provide value-based care.^25^ Implant selection is a chance to decrease costs to patients and maximize value of treatment. As surgeons integrate the concept of value into their practice, it can empower them to make cost-conscious decisions without compromising patient care.

This study had a number of strengths and weaknesses. A strength of this study is the use of PRWE as the PRO measure because it has been shown to have good responsiveness, validity, and reliability in the setting of DRFs.^22^ According to the best of the authors' knowledge, this is the first study to analyze the relationship between implant costs and patient outcomes in the setting of DRFs. This study is not without limitations. Inherent to the retrospective cohort design, this study only allows for the detection of correlation, not causation, between cost and outcomes. Three hundred fifty four were ineligible for analysis because of the lack of PROs or other injury and treatment characteristics. The study design and notable exclusion of patients create a potential for selection bias. Patients generally continue to improve up to a year after surgery, but this study only includes PRO scores up to 3 months of follow-up which is not representative of their final outcome. This may minimize the reported benefits of some of the more expensive VLPs (reduction of tendon irritation) that may not be recognized this early in recovery.^26^ Implant costs calculated in this study relied on the accuracy of patient charting. This study was conducted in a single healthcare system, which can limit the generalizability of these results. Finally, implant prices vary for each healthcare system due to the nature of contract negotiations between vendors, impacting the ability to directly compare with the prices in this study.

PROs for patients undergoing ORIF of DRFs using a VLP did not correlate with total cost of implants used. Optimal modality of treatment for DRFs is currently debated, but when surgeons use a VLP as the method of fixation, patients may not receive any additional benefit from more costly implants (e.g., variable angle plates and locking screws). As surgeons become more aligned with the principles of value-based healthcare delivery, they can take an active role as stewards over cost-effective care.
